# Limit-setting in online gambling: a comparative policy review of European approaches

**DOI:** 10.1186/s12954-024-01150-3

**Published:** 2025-02-13

**Authors:** Virve Marionneau, Elli Luoma, Tobias Turowski, Tobias Hayer

**Affiliations:** 1 Faculty of Social Sciences, Centre for Research on Addiction, Control, and Governance, University of Helsinki, Helsinki, Finland; 2 Department for Health and Society, Institute of Public Health and Nursing Research, University of Bremen, Bremen, Germany

**Keywords:** Gambling, Pre-commitment, Limit-setting, Europe, Policy

## Abstract

**Background:**

Online gambling products involve a heightened risk of harm, but there are some possibilities to prevent and reduce these harms. Notably, mandatory identification in online gambling environments allows for a range of pre-commitment tools such as limit-setting. Previous research has found that limit-setting tools can be helpful, but effectiveness depends on how policies are outlined and implemented. Limits can be financial or temporal, voluntary or mandatory, and system-level or operator-based. The current paper presents a policy review of European approaches to limit-setting in online environments.

**Methods:**

We first compared legal provisions on pre-commitment and limit-setting in N = 30 European countries (27 European Union Member States, Great Britain, Norway, and Switzerland). Data were retrieved from Vixio Gambling Compliance country reports and verified against original legal texts. The analysis focused on financial, temporal, maximum wager limits, and any other limits pertaining to online gambling. Second, based on the policy review, we produced a more in-depth analysis of limit-setting provisions in countries with system-level pre-commitment (Finland, Norway, Germany).

**Results:**

Results show important divergence in terms of limit-setting across Europe. While almost all countries (n = 27) have some form of limit-setting, implementation details vary. Financial limits can include loss limits (n = 15 countries), deposit limits (n = 18), and wagering limits (n = 14), with the majority of countries providing several types of financial limits. Temporal limits were available in ten countries. Eleven countries had some mandatory limits, in other countries operators were expected to provide the option to set limits. Statutory maximum limits and lower limits for young adults were not common, but available in some countries. Germany was the only country with a system-level limit-setting scheme that covered multiple licensed operators.

**Conclusions:**

Contextual variations in pre-commitment and limit-setting policies are likely to impact effectiveness in terms of preventing and reducing harm. Our review shows some promising practices, including system-level regimes, mandatory policies, reasonable maximum caps on limits and wagers, the possibility to set limits for various time periods, lower limits for young adults, and coupling limit-setting with other duty-of-care obligations. Learning from other jurisdictions can constitute good practice for future policies on pre-commitment.

## Background

Prevention and reduction of the complex harms from gambling requires a multi-level approach. From a public health perspective, universal methods should be prioritised. However, selective and indicated interventions are also needed [23, 39]. The digitalisation of gambling requires rethinking of many tried and tested interventions. Online gambling is characterised by constant availability and highly targeted offers, making universal restrictions on exposure and availability less straightforward to implement [25, 33]. In addition, online gambling can heighten harmfulness of many product-related characteristics. The possibility to play anonymously, the use of cashless payment methods, high event frequency, and the option to participate in several gambling products at the same time, may further increase the risk of gambling-related problems and harm [11]. A recent meta-analysis of problematic gambling globally found that online casino products and sports betting were amongst the most problematic product types for consumers [36].

From a consumer protection perspective, a key benefit of online environments is the ability to track consumption using behavioural data. Tracking data on consumers contain information on certain parameters of gambling behaviour such as money spent gambling (e.g., stakes, deposits), time spent gambling, or the accumulation of wins and losses per session or across time [9]. To enable better tracking, licensing conditions for online gambling operation generally mandate identification, often coupled with voluntary or mandatory pre-commitment measures [38].

Pre-commitment refers to a set of player-centric ‘responsible gambling’ tools that aim at facilitating individual control and consumer sovereignty [5]. Alongside consumption history reports and self-exclusion, key pre-commitment tools include limits on amounts of money and time spent gambling [27]. From the perspective of gambling providers, mandatory identification in online environments facilitates compliance with legislation on limit-setting. From the perspective of consumers, mandatory identification and limit-setting can enable better control over gambling and reduce the risk of harm.

A body of prior research has investigated the effectiveness of limit-setting, particularly from the perspectives of adoption, effects on consumption, and customer satisfaction. Overall, research has shown that pre-commitment systems are associated with positive effects in terms of addiction prevention [20]. However, when limit-setting regimes are voluntary, adoption rates vary significantly. An overview of studies [8] found that rates of adoption ranged from around 1 percent to up to 90 percent across samples. Mandatory limits are likely to be more effective as they are adopted by the full gambling population [8].

Several empirical studies have found that limits can reduce money and time spent gambling for those who do set them (e.g., [2, 4, 31]), although there is no available research on longer term effects [22]. Those with problematic gambling are more likely to set limits if these are voluntary. Individuals with problematic gambling also set higher limits than those who were not categorised as having gambling-related problems [27]. Conversely, Ivanova et al. [21] have shown that the increase or removal of personal limits can represent a risk marker for gambling problems.

A review by McMahon et al. [27] found that in half of the reviewed studies, individuals reported continued gambling after limits had been reached. This review was based mostly on studies reporting results of voluntary limit-setting. Moreover, a recent study using online gambling tracking data from Canada [41] demonstrated that individuals who set binding (i.e., unsurpassable) limits reduced their spending on gambling more than those who set non-binding limits. If limit-setting is voluntary, individuals may continue gambling by deleting or changing the limits. Voluntary limits can be seen as a tool for reflective motivation rather than for limiting physical opportunity to gamble [27]: For some, the possibility to set limits can function as an informational tool or as a motivation to seek help [26].

Overall, research evidence around limit-setting has been relatively patchy, with different outcome variables and differing contextual settings (e.g., [15, 20]). Implementation details are likely to have an important impact on the success and efficacy of any measure, because micro-level calibrations affect policy processes [7]. Prior research on limit-setting has drawn evidence from different country and company contexts, with varying practices and thresholds. Limit-setting can target monetary consumption but also time spent gambling. Monetary limits can be applied to deposits to gambling accounts, to bet sizes, or to total losses over a period of time [3]. In some cases, limits are reinforced by pop-up reminders (e.g., [14]). Practices also vary across operators. Without prescriptive maximum caps on limits, gambling operators have allowed widely differing maximum limits, reaching millions of Euros per month [24].

Understanding the contextual variations and calibrations in limit-setting policies is crucially important to improve regulations and their effects on gambling behaviours and harms. The current paper presents a review of European policy approaches to limit-setting in online environments. The aim is to compare what kind of thresholds and practices are available in Europe, and to identify good practices and recommendations for improved harm prevention and harm reduction in online gambling.

## Methods

### Data

We collected data on legal provisions for pre-commitment in 30 countries, including 27 European Union (EU) Member States as well as Norway, Switzerland, and Great Britain (gambling regulation is a devolved matter in Northern Ireland. Northern Ireland was not included). The choice to focus on these countries was motivated by data availability as well as maturity of gambling markets and regulations in this geographical area [38].

We first collected data from the Vixio Gambling Compliance database. Vixio Gambling Compliance is a gambling data intelligence provider, focusing on market and regulatory developments globally. The database is available under license. Vixio Gambling Compliance provides access to country profiles with summaries of the main legal provisions governing gambling within their jurisdiction. These profiles include information on ‘responsible gambling’ measures, such as limit-setting. In addition, Vixio Gambling Compliance provides online gambling specific information on legal requirements at country-level. VM and EL collected data on each jurisdiction, focusing on provisions on pre-commitment tools and limit-setting. We excluded self-exclusion policies and any limits pertaining to land-based gambling only from the analysis.

All data were collected in August and September 2024, reflecting the legal situation at that time. Data for the Netherlands were updated in October 2024 following reported changes to pre-commitment legislation on October 1st [18]. Vixio Gambling Compliance provided access to country profiles for all countries included, as well as providing references to original legal documents. All data included in our analysis were double checked against the original legal sources by the research team either in the original language, in an official English translation, or when translations were not available, using Google Translate. For ease of comparison, any sums given in other currencies than Euros are also provided in Euros, using an online currency converter [40] and the currency exchange rates of September 20th, 2024.

Second, we conducted a more in-depth case study analysis of countries with system-level pre-commitment policies (Germany, Finland, Norway). For this, we retrieved original legislative documents (Gambling Acts and Gambling Regulations documentation outlining pre-commitment practices and requirements).

### Data extraction

We produced a comparative policy review of legal provisions for limit-setting in 30 European countries. The analysis focused on financial (e.g., losses, deposits, and wagers) limits, temporal limits, maximum stake limits, and any other limits. Loss limits refer to the total sum that an individual can lose gambling within a specific period. Deposit limits represent the total sum that an individual can deposit on their gambling accounts within a specific period. Wager limits depict the total sum that individuals can bet within a specific period, including single bets. We also noted whether limits were system-level (governing the full gambling field) or operator-specific. To extract and classify data, we built a data template with Microsoft Excel.

Our focus on financial and temporal limits was motivated by existing recommendations in EU online gambling markets. The European Union has not harmonised its approach to gambling regulation, giving important leeway to Member States to regulate gambling markets as they best see fit. However, the European Commission has issued recommendations for improved consumer protection in online environments [12]. The document outlines key recommendations for pre-commitment. These include ensuring that upon registration, customers to gambling websites should be able to set monetary and temporal limits. Member States should also ensure that customers can reduce limits with immediate effect while the increasing of limits should come into effect twenty-four hours after the request at the earliest.

The more in-depth analysis of countries with system-level limit-setting provisions was conducted by inductive coding of retrieved legal documents based on how system-level limit-setting was technically executed and what kind of legislative framings and practices these systems were based on. The regulatory choices were compared and assessed within the framework of technical possibilities and the recommendations of the European Commission [12]. In line with literature on micro-level policy calibrations, this analysis aimed at analysing the concrete means used to achieve policy goals [7].

## Results

### European policy review

European jurisdictions share many similarities, but also differ in terms of their approaches to pre-commitment policies in gambling. A summary of the results of the legal provisions in 30 European countries is provided in Table 1.

**Table 1:** European policy approaches to pre-commitment in online gambling (N = 30)

Country	Loss limits	Deposit limits	Wagering limits	Temporal limits	Maximum Stake	Other limits
Austria	Varying state-level practices for betting.	Varying state-level practices for betting. For online casino products, mandatory maximum €400 (weekly) for over 26-year-olds, and €250 (weekly) for under 26-year-olds.	Varying state-level practices for betting.	Varying state-level practices for betting.	€500 (online gambling; state laws)	Higher deposit limits for online casino products with application for over 26-year-olds.
Belgium	None	Operator-based mandatory maximum €200 (weekly)	None	None	None	None
Bulgaria	None	None	None	None	Operator-based	None
Croatia	Operator-based option	None	None	None	None	None
Cyprus	Operator-based option	Operator-based option	None	None	Operator-based	None
Czech Republic	Operator-based option (daily and monthly)	Card tournaments maximum CZK 1,000 (€39.86) per 24 h	Operator-based option (daily and monthly)	Operator-based option (logins and time spent monthly)	CZK 500 (€19.93) (online gambling)	None
Denmark	None	Mandatory (daily, weekly, monthly)	None	None	None	None
Estonia	Operator-based option (weekly and monthly)	None	None	None	None	None
Finland	Mandatory maximum €500 (daily) and €2,000 (monthly) (all fast-paced products)	Mandatory (daily and monthly)	None	None, but time reminder every 60 min	Product-specific limits for online EGMs and casino products.	No deposits between midnight and 6am; Maximum amount on gambling account €20,000
France	No	Mandatory (weekly)	Mandatory (weekly)	Mandatory (poker only) with warnings	None	Withdrawal threshold (mandatory)
Germany	Operator-based option (daily, weekly, monthly)	Mandatory maximum €1,000 (monthly, cross-provider online); Additional optional operator-based option (daily, weekly, monthly)	Operator-based option (daily, weekly, monthly)	None, but mandatory 5-min breaks every 60 min (online EGMs)	€1 (online EGMs)	None
Great Britain	Operator-based optional (daily, weekly, monthly)	Operator-based optional (daily, weekly, monthly)	Operator-based optional (daily, weekly, monthly)	None	£2 (€2.38) for 18–24-year-olds; £5 (€5.96) for over 25-year-olds. (online EGMs)	Requirement to provide facilities to assist customers in sticking to their personal budgets
Greece	None	Operator-based option (daily, weekly, monthly)	None	Mandatory (daily, weekly, monthly)	€20 (online products ‘conducted using random number generators’ (RNG)	None
Hungary	Operator-based option	Operator-based option	Operator-based option	Operator-based option	None	None
Ireland (Republic of)	None	None	None	None	None	None
Italy	Operator-based option/mandatory	Operator-based mandatory (weekly)	None	Operator-based option	Operator-based	Limitations based on consumer profile
Latvia	None	None	Operator-based option for maximum total bets (daily)	None	Operator-based	Additional limits provided in industry code of conduct
Lithuania	None	None	Operator-based option for maximum total bets (time period or session)	None	Operator-based	None
Luxembourg	None	None	None	None	None	None
Malta	Operator-based option	Operator-based option	Operator-based option	Operator-based option (time and session limits)	None	None
Netherlands	Operator-based mandatory (daily, weekly, monthly)	Operator-based mandatory maximum. Existing customers: €700 per month for over 25-year-olds, €300 per month for 18–25-year-olds; New customers: €350 for over 25-year-olds and €150 for 18–25-year-olds	Weekly wager limit €100 for under 24-year-olds, €1,000 over 24-year-olds (betting offered by TOTO)	Operator-based mandatory (daily, weekly, monthly); limit reminders every 30 min	Horse racing maximum stake €250 single bets or €1,000 combined bets	If set deposit limit is over €350 (or €150 for 18–25-year-olds), displayed warning text and helpline number
Norway	Mandatory maximum NOK 20,000 (€1,703.83) (Norsk Tipping and Norsk Rikstoto); Online casino mandatory maximum NOK 4,000 (€340.77) (daily) and NOK 7,500 (€638.94) (monthly)	Lower monthly limits for 18-20 year-old at Norsk Tipping	Mandatory wager limit NOK 20,000 (€1,703.83) (daily and monthly)	Online casino mandatory time limit; 15-min mandatory break after 1 h of gambling	EGMs maximum NOK 100 (€8.52) (or ten times speed in seconds); Table games maximum NOK 500 (€42.60) but first bet maximum NOK 100 (€8.52)	None
Poland	None	None	None	None	None	General requirement for online gambling operators to provide mechanisms allowing customers to control their gambling
Portugal	None	Operator-based optional (daily, weekly, monthly)	Operator-based optional (daily, weekly, monthly)	None	Operator-based	None
Romania	None	Operator-based optional (daily, weekly, monthly); Maximum €200 deposits upon first registration	None	No, but reminder after every 60 min	None	None
Slovakia	Operator-based optional (monthly upon every login)	None	Operator-based optional (monthly upon every login)	None	None	None
Slovenia	None	None	Operator-based optional (session, period of time, or gambling product-specific)	None	None	None
Spain	None	Operator-based mandatory for online casino-type products (session-specific)	None	Mandatory for online casino-type products (session-specific); Reminders every 60 min	None	‘Intensive players’ prevented from using credit cards
Sweden	Operator-based mandatory (daily, weekly, monthly	Operator-based mandatory (daily, weekly, monthly)	None	Operator-based optional	None	None
Switzerland	Operator-based mandatory (daily, weekly, monthly)	None	Operator-based mandatory (daily, weekly, monthly)	None	None	For ‘large-scale’ gambling, limits can be waived

The results show that almost all European countries (n = 27/30) have some kind of legal provisions for limits on online gambling. In three countries (i.e. Republic of Ireland, Luxembourg, Poland), a pre-commitment approach or specific pre-commitment elements appear to be completely non-existent, at least at the level of legal provisions. Operator-based policies may nevertheless exist.

The types of financial limit-setting options available to consumers are quite evenly split, with provisions for loss limits in 15 countries, deposit limits in 18 countries, and wagering limits in 14 countries. Temporal limits are less common, with one third of jurisdictions (n = 10) having legal provisions for this type of limit-setting. Two jurisdictions (Germany, Norway) had provisions for mandatory breaks in gambling, with five other countries mandating warnings or reminders at predefined intervals (Finland, France, Netherlands, Romania, Spain). Almost half of the countries reviewed (n = 14) had some policy regarding maximum stakes for online gambling products.

In all countries except for Germany, limit-setting was operator-based. However, as Finland and Norway operate state monopolies in their online gambling provision, in these countries, limit-setting also covered the full legal online gambling market. Similarly, in Austria, online casino products are operated by a state monopoly and limits cover the full legal casino product provision. Online betting is regulated at state-levels with varying licensing practices. In other countries, pre-commitment, when available, was operator-based. In eight countries (Belgium, Denmark, France, Italy, Netherlands, Spain, Sweden, Switzerland) operator-based limits were mandatory, although maximums were not always provided. In these countries, consumers needed to set limits in order to access gambling.

In the remaining countries, legal provisions mandated operators to allow customers to set limits, but this was not a precondition for accessing gambling products. For example, in Cyprus, the law states that customers must be allowed to set individual maximum betting, loss, and time limits (Article 67(h), Cyprus Betting Law [44]). In Estonia, licensed providers must, before admitting customers to play for the first time ‘offer in an attention-grabbing manner the possibility to set an upper limit on the amount that the player is willing to lose in a day or month from gambling at the operator’ (Sect. 55(1) of the Estonian Gambling Act) [46].

Statutory maximums for financial limits were provided in seven countries. However, these limits varied in terms of technical, monetary, or temporal frameworks. A maximum cap for deposit limits was available in Austria (casino products only; €400 per week for over 26-year-olds with possibility to increase limit with application, €250 per week for under 26-year-olds), Belgium (€200 per week), the Netherlands (€700 per month for over 25-year-old and €300 per month for 18–25-year-old existing customers; €350 per month for over 25-year-old and €150 per month for 18–25-year-old new customers), and Germany (€1,000 per month). In Belgium and the Netherlands, these limits were operator-specific, whereas in Germany deposit limits were cross-provider [42, 50]. In Italy, operators need to consider the age and gambling behaviour of a customer in their determination of maximum deposit limits (Sect. 15(1)(b) of the Italian Legislative Decree of 25 March 2024, n. 41). In the Czech Republic, a maximum deposit limit of CZK 1,000 (€39.85) per 24 h applied to card tournaments only (Sect. 64 of the Czech Republic’s Gambling Act) [45]. In Romania, a €200 maximum deposit limit applies to the first deposit upon registration. However, the aim of the Romanian limit relates to player data verification procedures rather than responsibility measures (Articles 129, 130, and 132 of the Romanian Gambling Regulation).

Maximum loss limits were provided only in the monopolistic countries of Finland and Norway. In Finland, all ‘fast-paced’ online gambling was limited by a mandatory maximum loss limit of €500 a day and €2,000 a month (Decree VN/26957/2021). In Norway, the total maximum loss limit for both monopoly holders (Norsk Tipping and Norsk Rikstoto; separately) was NOK 20,000 (€1703.83) per month. Norsk Tipping applies a lower monthly loss limit to 18-20 year-olds but this is a company policy. Norway also had maximum loss limits for separate product categories. A daily NOK 4,000 (€340.77) limit and a monthly NOK 7,500 (€638.94) maximum limit applied to online casino gambling (Sects. 26 and 28, Norway’s Gambling Regulations). Norway also had a mandatory maximum wagering limit of NOK 20,000 (€1,703.83 euros) applied both daily and monthly. In the Netherlands, a similar mandatory maximum limit only applied to one national provider and depended on the age of the customer (see Table 1 above).

In other countries with limit-setting provisions, maximum amounts were not decreed by law. In these cases, operators can freely choose the kind of limit options given. Furthermore, in countries such as Italy and Great Britain, the law required some forms of financial limits, but did not specify if these should be based on losses, deposits, or wagers. In Poland, the law required ‘mechanisms allowing customers to control their gambling’ but did not specify whether this meant limit-setting (Poland Gambling Act, 2022: Article 15i).

Countries also differed in terms of how they adjusted time periods for different limits. For example, in France, all weekly limits concerned the ‘running seven-day period’ (Decree 2010–518, 2010: article 16) [49]. In Greece, all limits concerned the calendar day, week, or month (Greece Ministerial Decision No. 79835/2020: Article 24.2) [51]. In Spain, customers must set a maximum time and deposit limit each time before commencing gambling on casino-type products (Spain’s Royal Decree 176/2023: Article 13(3)). In Slovenia and Slovakia, operators must present the opportunity to set limits for the session or other periods upon every login (Sect. 32(1)(g) and (3) of Slovakia’s Gambling Act; Article 16 of Slovenia’s rules on organising games of chance via the Internet or other means of telecommunication).

Seven jurisdictions had provisions for mandatory maximum stakes for some gambling products. In most cases, these concerned online EGMs, with maximum stakes ranging from €500 in Austria to one euro in Germany. In Norway, the maximum stake for EGMs (including online EGMs) was defined as’ten times the speed of the product in seconds’ (e.g., if the event frequency of a product is, for example, 5 s, the maximum stake can be NOK 50). However, stakes cannot exceed 100 NOK (€8.52) in any case (Sects. 26 and 28, Norway’s Gambling Regulations). In other countries, maximum stakes may be gambling product or provider specific and not decreed by law. For example, in Bulgaria, operators are only obliged to display minimum and maximum stake sizes for different products (Article 17(3) of Bulgaria’s Decree No.31, 2021 [43]). In Portugal, licensed concessionaries must fix the minimum and maximum wager limits based on certain percentage rules and communicate these to the regulator (Article 58 of Portugal’s Decree-Law No. 422/89). In Lithuania, operators are to allow customers to set their own maximum limits for individual bets (Article 20^6^(1)(1) and 20^6^(1)(2) of the Lithuanian Gaming Law).

Three countries had separate provisions for limit-setting for younger consumer groups. In the Netherlands, operator-based mandatory maximum deposit limits were lower for 18–25-year-olds (€300, in comparison to €700 for over 25-year-olds). Furthermore, betting products provided under the state-owned operator were subject to lower weekly wagering limits for under 24-year-olds (€100) than other customers (€1,000; Article A.1(c) of the Netherlands’ Licence for sports betting; [18]). In Austria, deposit limits in online casino gambling were lower for under 26-year-olds (€250 per week) than for over 26-year-olds (€400 per week), with only those over 26 years of age were able to request a higher maximum limit. In Great Britain, a new stake limit implemented in September 2024 applies to online EGMs. For 18–24-year-olds, the maximum stake is two pounds, while for over 25-year-olds the maximum stake is five pounds [37]. Furthermore, in Italy, operators need to consider the age of customers in predefining limits (Italian Legislative Decree of 25 March 2024, n. 41) [52].

Some countries also had provisions for situations in which a customer wants to either raise or lower their limits. In all countries, lowering personal pre-commitment limits took place immediately or as soon as possible, but raising limits involved waiting times of different durations, ranging from 24 h to seven days (Italy and Germany). In Sweden, statutory duty of care provisions mandated that if a player raises their limit or sets a limit higher than SEK 10,000 (€877.84) a month, the operator should contact the individual (Chapter 11, Sect. 5 of Sweden’s Gambling Ordinance). Similarly, in the Netherlands, net deposits over €700 for over 25-year-olds or over €300 for 18–25-year-olds are considered possible markers of harm in line with duty-of-care obligations [55].

Individual countries had also introduced other types of pre-commitment tools. In Finland, deposits are disabled between midnight and 6am (Decree 27.04.2022 VN/26957/2021) [48]. France and Finland had provisions for maximum amounts on accounts. In Finland, this maximum was set at €20,000 (Decree 27.04.2022 VN/26957/2021) 48. In France, customers of licensed gambling sites were obligated to set an automatic transfer limit. When the account balance reaches this level, all funds exceeding the limit are automatically transferred to an associated bank account (Decree 2010–518 of May 19th, 2010: art. 17).

### System-level pre-commitment policies

Only three European countries (Finland, Norway, Germany) had a system-level approach to pre-commitment. In the following, we analyse the provisions in these countries in more detail.

#### Finland and Norway

In Finland and Norway, system-level limit-setting was enabled by the existence of monopolistic provision which included mandatory pre-commitment. In both Finland and Norway, online and land-based gambling require mandatory identification as well as limit-setting, covering most products. This is enabled by a single account for all gambling in Finland, and a near single account in Norway (the Norwegian market is split between two monopolies but Norsk Tipping controls most market segments).

In Finland, gambling regulation stipulates that one aim of the law is, among others, to prevent and reduce economic, social, and health harms (§1 of the Finnish Lotteries Act, Decree 1047/2001) [47]. Limit-setting provisions are available in The Ministry of the Interior’s decree on the gambling rules of monopoly-holder Veikkaus (Decree VN/26957/2021) [13]. The document outlines mandatory monthly and daily deposit limits, stating that ‘upon registration or at the latest before the first money transfer, a player has to set limits’ (Decree VN/26957/2021: 1). Deposit limits are binding for all products while loss limits concern ‘fast paced products’ (online EGMs, online bingo, online scratch cards, online table games, fast lotteries) (Decree VN/26957/2021: 4). A current proposed law is set to reform the regulation of online gambling in Finland in January 2027 (Finland’s Decree VN/27332/2023) [48]. The proposal is likely to change the situation in Finland towards an operator-based approach.

In Norway, the legislative framings require preventing gambling problems and other negative consequences, as well as maintaining a responsible and safe provision (§1 of the Norwegian Gambling Act). The pre-commitment policy is outlined in Sects. 26 and 27 of the Norwegian Gambling Regulations, requiring both monopoly holders to identify their customers and to mandate limit-setting. Loss limits of NOK 20,000 (1,703.83 euros) per month apply to both monopolies, with separate limits for certain products (see Table 1). However, winnings of up to NOK 20,000 (€1,703.83) can be included in the calculation of the loss limits.

#### Germany

Germany is the only country in Europe with binding system-level limits currently in place for a multi-operator market environment. The pre-commitment policies are legislated in the State Treaty on Gambling (GlüStV) [50] that came into force in July 2021. Before, online gambling was prohibited in Germany with the exception of the federal state of Schleswig–Holstein [19]. The licensed market currently (October 2024) includes sports betting (30 licenses), online EGMs (39 licenses), online poker (5 licenses), and online casino products (5 licences) [17]. 

The GlüStV [50] provides five objectives that regulations aim to fulfil. They include (1) ‘preventing the development of gambling and betting addiction’, (2) ‘ensure the protection of minors and gamblers’, and (3) ‘channel the natural gambling instinct of the population toward orderly and supervised channels’ to ‘counteract the development and proliferation of illicit games of chance in black markets’ (§1 (1), (3), and (2) of the GlüStV, respectively). The GlüStV [50] has provisions for pre-commitment in online gambling, including the monthly system-level deposit limit of €1,000. The limit is required upon registration for all licensed online offers, except for lotteries that are not held more than twice a week and for lotteries in the form of prize savings. On registration, customers are asked to set a monthly deposit limit, with no maximum amount shown in the prompt. The binding maximum amount should only be displayed if individuals attempt to specify a higher amount than €1,000 (as a request to correct the given amount) (§6c (1) of the GlüStV, [50]1).

The deposit limit registry is centrally managed by the Joint Gambling Authority (a so-called ‘limit file’; §6c (4) of the GlüStV [50]). Compliance with the limits is monitored using data stored in this limit file. In addition, players have the option of setting up operator-based daily, weekly, or monthly wager, deposit, and loss limits. Having the option to set these limits is mandatory (§6c (2) of the GlüStV [50]).

On a discretionary basis, the authority can grant increases to the maximum system-level deposit limit. Providers are also not allowed to advertise the option of an increased maximum deposit limit. These increases can be either up to €10,000 or up to €30,000. A limit higher than €1,000 comes with several requirements: (1) The deposit limit has to be set individually, (2) a loss limit of at maximum 20% of this individual new deposit limit has to be set, (3) an annual proof of financial standing, e. g. via income tax statements, has to be offered by the gambler, and (4) operators need to implement a special monitoring for conspicuous gambling behaviour, whose result needs to be transmitted in anonymized form to the Gambling Authority every six months.

An increase between €10,001 and €30,000 additionally requires a) at least 21 years of age, b) close monitoring of the individual gambling behaviour by the provider, the results of which need to be transmitted quarterly to the Gambling Authority, and c) if at-risk or addictive gambling behaviour is detected in monitoring, this needs to be reported immediately to the Gambling Authority who will then decide on measures taken (e.g., revoke the right to a higher limit or exclude the customer). The form of this close monitoring is not specified. A deposit limit between €10,001 and €30,000 is only allowed for 1% of the active gamblers for a given operator. A monthly deposit limit of over €30,000 is not allowed under any circumstances.

## Discussion

Limit-setting allows individuals to track and control their gambling. However, prior research has suggested that effectiveness of limit-setting regimes depends largely on implementation and calibrations (e.g., [8]). This policy review has focused on mapping what kind of approaches to limit-setting are available in European countries (N = 30) for online gambling. The results have shown that while most countries have some kind of policy related to limit-setting, limits are binding only in a minority of countries. The most common types of limits related to financial forms of pre-commitment, such as limits on deposits, losses, and wagers. However, the concrete specifications varied considerably between jurisdictions.

The European Commission [12] principles for the protection of consumers and players of online gambling services were visible in our results. While the Commission principles are not legally binding, many jurisdictions complied in terms of providing options for limit-setting, and by ensuring that increases in personal limits would not take effect until at least 24 h later – in many cases more. Similarly, a recent global review of legislative reforms in gambling [38] (N = 33), found that while voluntary pre-commitment systems were implemented across continents, mandatory pre-commitment systems were only found in European markets. It is possible that the European Commission principles have had an effect in advancing the regulatory environment across the continent. This suggests that even non-binding recommendations from important international bodies may have – among other sources of influences – an important impact on improving consumer protection measures in gambling.

From the perspective of reducing and preventing gambling-related harms, our review has highlighted several promising practices. Existing reviews into effective harm prevention policies in gambling lend support to universal and binding models [14, 39]. In our policy review, such models included system-level and cross-provider limit-setting regimes, mandatory policies with prescriptive maximum caps on limits, prescriptive maximum stake sizes for harmful gambling products such as online EGMs, and the possibility to set limits for various time periods, including session-specific limits, as well as daily, weekly, and monthly limits. Binding, rules-based implementation and enforcement of limit-setting reduces self-regulatory burden on individuals and gambling operators. As has also been argued by others (e.g., [16, 28, 29], a ‘responsible gambling’ framework highlighting individual responsibility can be stigmatising and harmful to consumers, but it can also divert attention away from more effective systemic solutions.

In addition, lower limits for special groups such as under 24-year-olds appear to be a good practice. Research shows that this demographic, and especially young males, are at a particular risk for problematic gambling, particularly in online environments [1, 10, 30]. Other operator-specific lower limits for younger individuals are also available, although these were not captured in our legal review. One such limit was implemented by Swedish government-owned Svenska Spel. As of 2022, 18–19-year-old customers of the provider have had a reduced deposit limit cap of SEK 1,000 (€87.78) per month. According to the company’s own estimate, the measure has helped reduce the proportion of individuals with potentially harmful gambling from 9 to 3 percent within this population [35].

Other vulnerable groups may also benefit from lower limits. One review focusing on such groups [34] found seven different vulnerable populations. In addition to young individuals, these included older adults, women, veterans, indigenous populations, prisoners, and individuals with high poverty. These types of other vulnerable groups were not addressed in the pre-commitment regimes of any European country, although in the Netherlands lower limits applied to new customers due to a policy change. Moreover, there are no defined limits that take into account the current income situation of individuals (e.g., a deposit limit of five or 10 percent of the monthly net income). This type of solution could expand on existing affordability checks that currently take place in some countries when individuals want to apply for increased limits.

A few countries (Germany, Italy, Netherlands, Sweden) mandated some kind of player behavioural tracking or interventions as part of their pre-commitment policies. In Sweden and the Netherlands, statutory duty of care practices mandated operators to contact individuals who set high limits or increased their limits. In Italy, operators needed to consider the age as well as gambling consumption of individuals in predetermining consumer-specific maximum limits. In Germany, operators were mandated to closely monitor the gambling behaviour of individuals who had been granted a discretionary higher deposit limit. In some other countries, companies used similar behavioural tracking as part of their own responsibility programmes.

These kinds of emerging practices are in line with the ‘know your customer’ obligations for operators, and can enable harm prevention alongside other obligations, for example in anti-money laundering (e.g., [32, 37]). These types of approaches should be developed further to improve consumer protection. However, responsibility for these types of data-driven practices should not be left solely to the discretion of gambling operators. In Germany for example, oversight over implementation and enforcement is closely monitored by the Gambling Authority. This is likely to improve compliance, but also highlight the importance of regulatory authority in addressing gambling harm. 

Individual policy tools, such as limit-setting or behavioural tracking should not be seen as stand-alone solutions but as parts of a wider policy instrument mix. Policy mixes are in many cases complementary and enhance overall protections [6]. The implementation of several complementary pre-commitment and harm reduction measures is therefore likely to result in better outcomes from the perspective of consumer protection. 

### Limitations

This study has some limitations. We have reviewed pre-commitment and limit-setting policies in 30 European countries. The countries selected for the analysis represent jurisdictions within the European Union or other high-income and mature gambling markets within the area. The choice to focus on only these countries was partly necessary due to issues of data availability. It is likely that the inclusion of a larger geographical area would have changed the results of the policy review.

In addition, the first round of data collection was conducted using the database of Vixio Gambling Compliance. While the database aims to be up to date, it may be limited with regard to information on some jurisdictions. We double-checked all data against original legal documents, but it is possible that some documents were not identified. It should also be noted that legal requirements do not always correspond to implementation in practice and possible circumventions could not be documented here (e.g., the possibility that individuals may have more than one player account). Company or industry trade association self-regulatory policies were also not included. These are likely to have had additional measures available. Furthermore, our inability to read all European languages meant that, in some cases, we needed to resort to unofficial English translations or Google Translate. This may have resulted in some details or nuance missing from the analysis.

Finally, the regulation of online gambling is a constantly changing space. For example, the Republic of Ireland is currently processing a new Gambling Regulation Bill, proposing new limits on bets. Spain is also currently in the process of introducing a system-level limit-setting regime. The current analysis was able to provide a picture of limit-setting policies in autumn 2024, but we were not able to assess all on-going legal processes.

Further studies should focus particularly on assessing the effects of different limit-setting regimes. Contextual factors and details related to the implementation policies are likely to have an important impact on how effective limit-setting can be in terms of reducing harmful gambling consumption. Different policy mixes may also result in different outcomes. Currently, little policy evaluation literature is available with regard to limit-setting, particularly in online environments. Particularly, evaluation of the German pre-commitment system would be important to provide advice also to other European countries, as it is currently the only non-monopoly system with binding cross-provider regulation. In addition, it would be important to investigate the policy processes and influence (e.g., research findings, political negotiation processes, anecdotal evidence) involved in differing choices in terms of limits and pre-commitment.

## Conclusion

Limit-setting regimes are prevalent in online gambling environments in Europe. However, the implementation of these policies varies. The results of this paper have shown that few European countries operate mandatory limit-setting regimes with prescriptive maximum caps on limits. Only one country operates a cross-provider limit-setting policy in a competitive online market. Limit-setting can be an effective tool to improve the prevention and reduction of harm in online environments. However, the implementation context is likely to affect policy efficiency. Going forward, it is crucial that countries in Europe, and beyond, share good practices and evaluate policies to improve regulatory collaboration, harm reduction policies, and public health.

## Data Availability

Country profile data are available from Vixio Gambling Compliance under license. Legal documents are available in the public domain. These data supporting the analysis are also available from the authors.

## List of abbreviations

CZK: Czech koruna
EGM: Electronic gaming machine
EU: European union
GlüStV: Glücksspielstaatsvertrag/state treaty on gambling
NOK: Norwegian krone
SEK: Swedish krona
